# Regulation of Glucose Metabolism by NAD^+^ and ADP-Ribosylation

**DOI:** 10.3390/cells8080890

**Published:** 2019-08-13

**Authors:** Ann-Katrin Hopp, Patrick Grüter, Michael O. Hottiger

**Affiliations:** 1 Department of Molecular Mechanisms of Disease (DMMD), University of Zurich, CH-8057 Zurich, Switzerland; 2 Molecular Life Science Ph.D. Program, Life Science Zurich Graduate School, CH-8057 Zurich, Switzerland

**Keywords:** carbohydrate metabolism, ADP-ribosylation, ARTD, PARP, NAD^+^, NR, NMN, NAM

## Abstract

Cells constantly adapt their metabolic pathways to meet their energy needs and respond to nutrient availability. During the last two decades, it has become increasingly clear that NAD^+^, a coenzyme in redox reactions, also mediates several ubiquitous cell signaling processes. Protein ADP-ribosylation is a post-translational modification that uses NAD^+^ as a substrate and is best known as part of the genotoxic stress response. However, there is increasing evidence that NAD^+^-dependent ADP-ribosylation regulates other cellular processes, including metabolic pathways. In this review, we will describe the compartmentalized regulation of NAD^+^ biosynthesis, consumption, and regeneration with a particular focus on the role of ADP-ribosylation in the regulation of glucose metabolism in different cellular compartments.

## 1. Introduction

### 1.1. NAD^+^ in Redox Reactions

Metabolic pathways are tightly regulated on various levels, which include co-enzyme availability, allosteric regulation with various metabolic intermediates, and post-translational modification of metabolic enzymes. Nicotinamide adenine dinucleotide (NAD^+^, in its oxidized state) is a vital small molecule, best known as a cofactor that regulates metabolism through its electron transfer function in redox reactions that regulate glycolysis, tricarboxylic acid (TCA) cycle, and oxidative phosphorylation driven energy metabolism [1,2,3]. In redox reactions, NAD^+^ is reversibly reduced to NADH [4]. NAD^+^ can be regenerated in the cytoplasm from NADH through the conversion of pyruvate to lactate by the lactate dehydrogenase (LDH), thereby maintaining active aerobic glycolysis [5]. Alternatively, electrons from NADH can enter the mitochondrial electron transport chain via the glycerol-3-phosphate or the malate-aspartate shuttle [6,7]. Within the mitochondria, NAD^+^ plays a central role by carrying high-energy electrons and driving oxidative phosphorylation [8]. NAD^+^ accepts electrons from different sources and transfers them to complex I of the electron transport chain, which results in the generation of ATP. The maintenance of NAD^+^ levels, as well as the NAD^+^/NADH ratio, is crucial for mitochondrial homeostasis and ATP production.

### 1.2. Cellular NAD^+^ Pools

The in-vivo half-life of NAD^+^ varies between 15 min and 15 h depending on the tissue [9]. Decreased NAD^+^ levels dampen the activities of NAD(H)-dependent enzymes involved in oxidative phosphorylation, TCA cycle, and glycolysis, which lowers the ATP production [10]. Several approaches have been developed to try to quantify NAD^+^ availability in different subcellular compartments. Compartment-targeted expression of the catalytic domain of ADP-ribosyltransferase diphtheria toxin-like 1 (ARTD1, formerly called PARP1) has been used as a molecular detector of NAD^+^ because formation of poly-ADP-ribose, which can be detected by immunofluorescence, functions as a proxy for NAD^+^ availability [11,12]. In addition, NAD^+^ metabolite tracing methods have been developed that quantify NAD^+^ and NADH levels, and define how their ratios in different subcellular compartments under several stress conditions change [11,13,14,15]. Free NAD^+^ has been measured in almost all organelles, including the mitochondria, peroxisomes, the endoplasmic reticulum (ER), and the Golgi complex [11,12,13]. These measurements have revealed that steady state NAD^+^ concentrations, as well as changes in NAD^+^ concentrations in response to different stimuli, vary considerably between different subcellular compartments. Hence, NAD^+^ dynamics seem to be compartment specific. Furthermore, these dynamics also vary depending on the cell type, stress, and redox status as well as metabolic fitness [16]. The NAD^+^ concentrations are highest in the mitochondria (approx. 400 μM, 40%–70% of the total cellular NAD^+^ pool [17,18]), intermediate in the nucleus and cytoplasm (approx. 100 μM) and low (<1 μM) in the extracellular space [13,15,19]. Cytoplasmic and nuclear NAD^+^/NADH ratios are typically between 60 and 700 in eukaryotes depending on the cell type, while the mitochondrial ratio is much lower at around 7–8 [20,21]. Nuclear and cytoplasmic NAD^+^ pools are interconnected as NAD^+^ levels in both compartments are generally comparable and NAD^+^ precursors freely diffuse through the nuclear pore complex. In contrast, NAD^+^, NADH and NAD^+^ precursors cannot freely diffuse across mitochondrial membranes, and thus mitochondrial NAD^+^ pools are more isolated [22]. This compartmentalization of NAD^+^-levels provides a special level of regulation and sophistication that requires tightly regulated expression of enzymes involved in NAD^+^ biogenesis.

### 1.3. NAD^+^ Biogenesis/Synthesis

NAD^+^ is constantly synthesized, degraded, and recycled, not only in the cytoplasm where most research is focused, but also within major organelles including the nucleus, Golgi, and peroxisomes [23,24]. In mammals, NAD^+^ is generated either de-novo via the kynurenine pathway from tryptophan, via the Preiss-Handler pathway from nicotinic acid (NA), via the salvage pathway from nicotinamide (NAM), or via the nicotinamide ribose kinase pathway from nicotinamide riboside (NR) [25,26,27,28]. Many enzymes involved in the biosynthesis of NAD^+^ are highly compartmentalized on the subcellular level and tightly regulated on the transcriptional level, which provides the rationale as to why some tissues and/or cell types are able to synthesize NAD^+^ from certain sources and others are not. A detailed overview of the NAD^+^ metabolic pathways has been extensively described in other reviews [1,25,29,30].

In-vivo, the de-novo synthesis of NAD^+^ from tryptophan takes place predominantly in the liver. The liver then excretes NAM which is subsequently taken up by other organs and converted to NAD^+^. NAD^+^ fluxes vary widely across tissues, with high fluxes in the small intestine and spleen and low fluxes in skeletal muscle [9]. Intravenous administration of NR or NMN delivered NAD^+^ to multiple tissues, but the same agents given orally were metabolized to NAM, indicating that the type of administration and the involved organs metabolize NAD^+^ precursors differently [9]. In-depth analysis of NAD^+^ synthesis-breakdown fluxes in different cell lines revealed that the vast majority of immortalized cells lack the enzymes to synthesize NAD^+^ from tryptophan, and thus they depend entirely on NAM [9]. Consequently, most cell lines depend on two enzymes to generate NAD^+^. The first is nicotinamide phosphoribosyltransferase (NAMPT) which catalyzes the conversion of NAM to NMN; this is considered the rate limiting step of the NAD^+^ salvage pathway [31]. The second family of enzymes required are the nicotinamide mononucleotide adenylyltransferases (NMNATs), which convert NMN to NAD^+^. NAMPT is primarily nuclear and cytosolic; however, a small portion co-purifies with mitochondria isolated from the liver [32]. There are both intracellular and extracellular isoforms of NAMPT that are known as iNAMPT and eNAMPT, respectively [33]. Three isoforms of NMNAT have been identified; NMNAT1 localizes to the nucleus, NMNAT2 to the Golgi apparatus and neuronal axons, and NMNAT3 to the mitochondria [34,35,36]. Given that both NAMPT and NMNAT3 localize to the mitochondria, it has been suggested that mitochondria might recycle their own nicotinamide or take it up from the cytoplasm. Increasing evidence has revealed that (i) reduced NAD^+^ levels alter metabolic rates and increase age-related disease susceptibility and (ii) restoration of NAD^+^ levels can prevent disease progression [37,38]. 

### 1.4. NAD^+^ Signaling

Within a given location, NAD^+^ has two main pools, the *free* pool and *protein-associated* pool. Moreover, the ratio of these pools varies across different organelles, cell types, and tissues [29]. NAD^+^ is predicted to bind >500 proteins involved in the regulation of almost all major biological processes [39,40]. Two principal types of NAD^+^-dependent signaling reactions have recently been described: the generation of second messenger molecules and the modification of proteins [41,42]. NAD^+^ is a direct precursor of various small molecules such as ADP-ribose (ADPr), cyclic ADP-ribose (cADPr), NAADP and O-acetyl-ADP-ribose (OAADPR); all of which are important second messengers that regulate multiple aspects of biology, including cell survival, apoptosis, and inflammation [39,43,44,45,46,47,48]. In addition, NAD^+^ serves as a substrate for post-translational protein modifications (PTM). In mammals, the two main NAD^+^-dependent enzyme families are the ADP-ribosyltransferases (ARTs) and the sirtuins (SIRTs) [39,49,50,51,52]. Both protein families use NAD^+^ as a co-substrate to modify or de-modify target proteins. Interestingly, ADP-ribosylation was the first NAD^+^-dependent PTM identified [53]. ADP-ribosylation involves the attachment of one (mono-ADP-ribosylation; MARylation) or several (poly-ADP-ribosylation; PARylation) moieties of ADPr onto specific amino acid acceptor sites of target proteins or onto ribonucleotides [54]. Depending on their structural similarities to either cholera- or diphtheria toxins, ARTs can be further subdivided into the intracellular ARTDs (Diphteria toxin-like) and the extracellular or membrane-associated ARTCs (Cholera toxin-like). Despite the very low NAD^+^ concentrations in these compartments [55], ARTCs as well as cADPr hydrolases, such as CD38, can metabolize NAD^+^ in the extracellular space and in serum. Since this review focuses on the influence of ADP-ribosylation on carbohydrate metabolism the reader is referred to other reviews describing extracellular NAD^+^ consumption [45,56]. SIRTs remove acyl marks (most commonly acetylation) from proteins using NAD^+^ as an acceptor, thereby generating OAADPR and NAM [39,57]. SIRTs and ARTs localize to various intracellular compartments and have been experimentally linked to distinct cellular functions. Both enzyme families are associated with the regulation of various physiological processes, including metabolic regulation, DNA damage repair, cell cycle progression, and epithelial-to-mesenchymal transition (EMT) [58,59,60,61,62].

It is important to note that when functioning as a coenzyme, for instance in redox reactions, NAD^+^ is reversibly converted to NADH. In contrast, when NAD^+^ functions as a signaling molecule in NAD^+^-dependent signaling processes, NAD^+^ but not NADH, is used as a substrate [63] and is continuously and irreversibly catabolized by cleavage of the glycosidic bond between NAM and the ADPr moiety. In cells, the involvement of NAD^+^ in deacetylation reactions or ADP-ribosylation requires constant re-synthesis of NAD^+^ to avoid depletion of intracellular NAD^+^ pools, which is particularly challenging for rapidly dividing cells [9].

NAM and ADPr are the common products generated as a result of NAD^+^-dependent signaling conversions. In human cells, ADPr can be cleaved by several members of the Nudix hydrolase family [64]. Among these enzymes, NUDT9 appears to have the highest specificity towards ADPr generating AMP and ribose 5-phosphate [65,66,67]. The recycling of these degradation products would require three molecules of ATP to regenerate NAD^+^. Therefore, NAD^+^-dependent signaling events, especially PARylation, are rather energy consuming.

### 1.5. ADP-Ribosylation and Carbohydrate Metabolism

**Figure 1:**
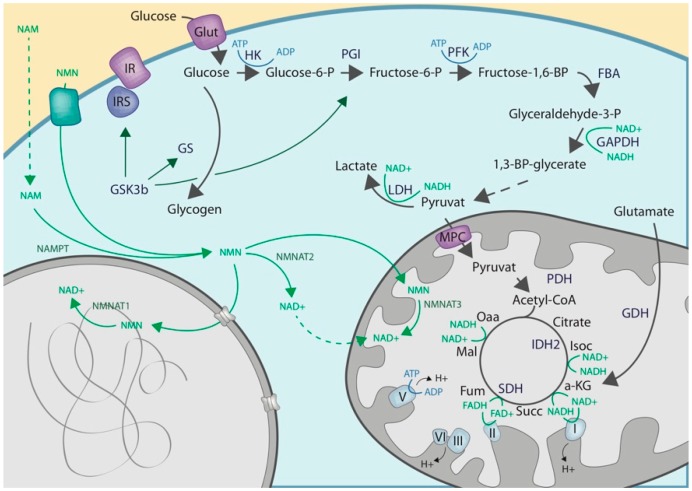
Schematic overview over the main metabolic hubs comprising the central carbohydrate metabolism. Gycolysis, TCA cycle and oxidative phosphorylation are depicted in black (black arrows), while enzymes involved in those processes are depicted in dark blue. NAD^+^ metabolism, including synthesis and consumption is depicted in green (green descriptions and arrows), while ADP/ATP conversions are depicted in light blue. Glucose-6-phosphate (Glucose-6-P), fructose-6-phosphate (Fructose-6-P), fructose-1,6-bisphosphate (Fructose-1,6-BP), glyceraldehyde-3-phosphate (Glyceraldehyde-3-P), oxaloactetate (OAA), isocitrate (Isoc), a-ketoglutarate (a-KG), succinate (Succ), fumarate (Fum), malate (Mal), hexokinase (HK), phosphoglucoisomerase (PGI, phosphofructokinase (PFK), aldolase (FBA), lactate dehydrogenase (LDH), pyruvate dehydrogenase (PDH), glutamate dehydrogenase (GDH), insulin receptor (IR), insulin receptor signaling (IRS).

ADP-ribosylation is considered energetically challenging for the cell, as each ADPr moiety attached to a given target protein requires the consumption of one NAD^+^ molecule. Therefore, ADPr-metabolizing enzymes such as ARTs and ADP-ribosylhydrolases (ARHs) have a very strong impact on intracellular NAD^+^ homeostasis [9]. Indeed, an anti-correlation between the activity of the predominant nuclear ART, ARTD1, and intracellular NAD^+^ levels has been the subject of various studies [68,69,70,71,72]. It is, thus, not surprising that ART activation has been linked to cell metabolism in various physiological and pathophysiological processes, including adipogenesis, genotoxicity-induced cell death, immune cell activation, and metabolic disorders [68,69,70,71,72,73]. In fact, the activity of these enzymes can influence glycolysis and oxidative phosphorylation directly and indirectly at various steps (see below). NAD^+^ and NADH are important redox-equivalents for various metabolic reactions, like the conversion of pyruvate to lactate or the conversion of succinate to fumarate and back (Figure 1). In addition, ADP and ATP are important co-factors as their ratio is strongly dependent on oxidative phosphorylation and, thus, NAD^+^. As a consequence, extensive ART activation decreases NAD^+^ and ATP levels, and indirectly influences anabolic and catabolic reactions involved in central carbohydrate metabolism. There is, however, growing evidence that ARTs also regulate the carbohydrate metabolism by directly modifying important regulators of the involved metabolic pathways. ADP-ribosylation has also been proposed to play a role in other metabolic processes, such as the lipid metabolism (reviewed in [74]). ADP-ribosylated proteins involved in glycose metabolism with assigned ADPr amino acid acceptor sites are summarized in Table 1.

## 2. Cytoplasmic Crosstalk of ADP-Ribosylation and the Carbohydrate Metabolism

### 2.1. Cytoplasmic NAD Biosynthesis 

Many enzymes involved in NAD^+^ biosynthesis, such as NAMPT and the NMNATs, and breakdown, like the ARTs and SIRTs, are highly compartmentalized on the subcellular level, providing yet another layer of NAD^+^ level regulation ([16]; Table 2).

The enzymes involved in the kynurenine and the Preiss-Handler pathway all localize to the cytoplasm. Therefore, de-novo NAD^+^ synthesis predominantly happens in this compartment. With NAMPT and NMNAT2 the cytoplasm also harbors a set of enzymes required for NAD^+^ recycling from NAM and NMN. As mentioned, for most transformed cells the salvage pathway is the main source of NAD^+^ synthesis. In those cells, the levels of NAMPT are highly dynamic and respond to changes in cellular demands for NAD^+^, such as starvation or severe DNA damage, for instance [32]. NMNAT2 catalyzes the final reaction, converting NMN to NAD^+^ in the cytoplasm. In the cytoplasm, NAD^+^ plays an important electron carrier role in the glycolysis, as it is required for the conversion of glyceraldehyde-3-phosphate to 1,3-biosphosphoglycerate, as well as for the conversion of pyruvate to lactate. In fact, reductions in cytoplasmic NAD^+^ levels are associated with reduced glycolysis and the necessity of cells to metabolize non-glucose carbon sources, such as glutamate or pyruvate via the TCA cycle [78]. 

### 2.2. Cytoplasmic ADP-Ribosylation

Many ARTD-family members, especially ARTDs catalyzing MARylation are described to localize to the cytoplasm (Table 2) [49]. Nonetheless, potential functions of ADP-ribosylation in the cytoplasm are rather poorly described. The best-studied ARTDs localizing to the cytoplasm are ARTD10, ARTD12, ARTD15 and the tankyrases (TNKs) [95,96,97,98,99,100]. ARTD10 and the TNKs have been linked to cytoplasmic metabolic processes, while the other cytoplasmic ARTDs have thus far not been directly associated with cell/carbohydrate metabolism. ARTD12 localizes to the Golgi and is involved in protein sorting and trafficking [95,96], while ARTD15 localizes to the ER and has been linked to the unfolded protein response [97,98]. For more detailed information about the function of other (cytoplasmic) ARTs, the reader is referred to other reviews [101]. Nothing has so far been reported regarding the regulation of the carbohydrate metabolism by cytoplasmic ARHs.

### 2.3. Crosstalk with Enzymes of the Carbohydrate Metabolisms

*Glut4*: Glut4 is one of 14 Glut transporters described in mammals [102]. Glut transporters are transmembrane proteins that are able to transport hexoses from the extracellular space into cells. Specifically, Glut4 transports glucose and glucosamine, and is predominantly expressed in adipocytes, skeletal muscle and cardiomyocytes [102,103]. While some glucose transporters, such as Glut1, constitutively localize to the cell membrane, Glut4 translocates to the plasma membrane in response to insulin stimulation [103]. Both TNK1 and TNK2 have been shown to influence Glut4 mediated glucose transport in an ADP-ribosylation-dependent manner [99,104]. While in adipocytes, *tnks* deficiency increased Glut4 levels, TNKs inhibition in myocytes destabilized Glut4 regulatory proteins, resulting in decreased Glut4 translocation to the plasma membrane [99,104]. However, in both studies *tnks* deficiency/inhibition affected insulin sensitivity. Furthermore, the use of TNK inhibitors supported the notion that this process is dependent on ADP-ribosylation; specific target proteins of TNK1/2, however, have not yet been identified. 

**Figure 2:**
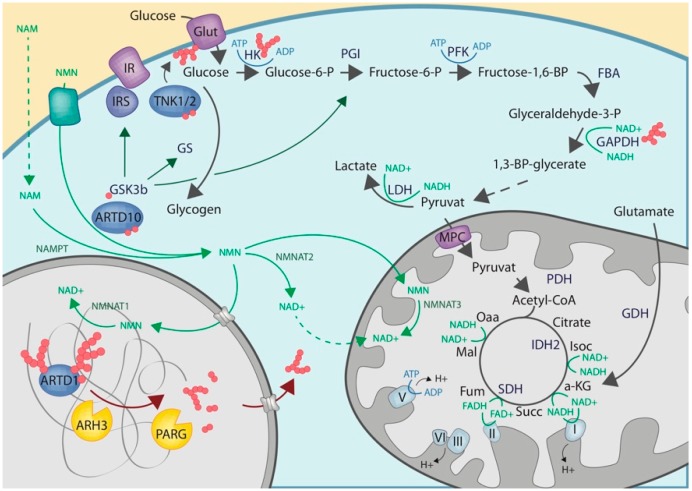
Schematic overview on the functional contribution of cytoplasmic ARTs to carbohydrate metabolism. Metabolic pathways are shown as in Figure 1. ADP-ribosyltransferases are depicted in blue, while ADP-ribosylhydrolases are depicted in yellow.

*Hexokinase 1*: Hexokinases catalyze the initial step of glycolysis by converting glucose to glucose-6-phosphate (Figure 2). In mammals, 4 isoenzymes, HK1, 2, 3, and 4 have been identified [105]. HK1 was shown to contain a PAR binding motif (PBM) that enables the enzyme to interact with PAR in a non-covalent manner [106]. Under normal conditions HK1 localizes to the mitochondrial membrane. Binding of HK1 to free cytoplasmic PAR, e.g., in the context of ARTD1 hyperactivation upon severe genotoxic stress, was proposed to lead to the segregation of HK1 from the mitochondria and to reduce its enzymatic activity in a dose-dependent manner, consequently slowing down glycolysis and oxidative phosphorylation [106]. This observation was strengthened by the observation that inhibition of PAR release by PARG knockdown rescued HK1 activity and the cells’ metabolic capacity. Together, the above-discussed study proposes a mechanism in which extensive nuclear ARTD1-mediated PAR formation results in a reduction in the enzymatic activity of HK1, which is independent on direct ADP-ribosylation, but mediated via PAR binding. 

*Glycogen synthase kinase 3b*: The glycogen synthase kinase 3b (GSK3b) has also been proposed to be regulated by ADP-ribosylation [100]. GSK3b is a serine-threonine-kinase that regulates glucose metabolism by controlling the activity of various enzymes involved in anabolic and catabolic carbohydrate metabolism, including the glycogen synthase, the insulin receptor, the glucose-6-phosphatase, and/or the phosphoenolpyruvate carboxykinase [107,108] (Figure 2). A recent study identified GSK3b as a direct ADP-ribosylation target of ARTD10 [100]. Intriguingly, studies also suggest that ARTD10 negatively regulates cell metabolism and mitochondrial function, as ARTD10 knockdown increased the respiratory capacities of various cell lines and reduced reactive oxygen species susceptibility via induction of antioxidant gene expression [109]. In addition, ARTD10 levels were also shown to change in response to fasting-induced metabolic stimulation [109]. A direct link between ARTD10-mediated ADP-ribosylation and cell metabolism, however, remains to be elucidated.

*Glyceraldehyde 3-phosphate dehydrogenase*: Another key-metabolic enzyme directly regulated via ADP-ribosylation is the glyceraldehyde 3-phosphate dehydrogenase (GAPDH). GAPDH possesses glyceraldehyde 3-phosphate dehydrogenase activity as well as nitrosylase activity. The enzyme plays an important role in glucose breakdown by converting glyceraldehyde-3-phosphate to 1,3-bisphosphoglycerate; a reaction that requires the reduction of NAD^+^ to NADH (Figure 1 and Figure 2). Since the second activity is linked to nuclear processes, the enzyme is considered a moonlighting enzyme [110,111]. Comparable to GSK3b, ADP-ribosylation of GAPDH has been shown to decrease its enzymatic activity. While initial studies suggested that GAPDH auto-ADP-ribosylates itself [112], more recent studies suggest GAPDH to rather be trans-ADP-ribosylated by either ARTD1 or ARTD10 [113,114]. ARTD1-mediated inhibition of GAPDH has been demonstrated in the context of hyperglycemia, where increased glucose concentrations in the cell culture medium resulted in increased DNA double-strand breaks as a consequence of an increase in mitochondrial superoxide production [113]. GAPDH inhibition could further be associated with an increase in PKC activity, as well as NF-κB activation; both of which are likely indirect responses to the increase in DNA-damage. In addition, increased metabolic flux into the hexosamine pathway was also observed as a possible consequence to reduced glycolysis rates [113]. Decreasing mitochondrial superoxide production (e.g., via overexpression of UCP-1 or mtSOD2) rescued all of the above-described consequences of hyperglycemia. Besides ARTD1, also ARTD10 was shown to interact with GAPDH [114]. Overexpression of full-length ARTD10 or its catalytic domain lead to an ADP-ribosylation-dependent sequestering of GAPDH in membrane-free, cytoplasmic cell bodies [114]. The functional consequence of this ARTD10-mediated ADP-ribosylation, as well as compartmentalization of GAPDH, has however so far not been further investigated. 

## 3. Mitochondrial NAD Biosynthesis, ADP-Ribosylation Crosstalk and Carbohydrate Metabolism

### 3.1. Mitochondrial NAD Biosynthesis

Supplementing cell culture medium with NAD^+^ or NR has been shown to increase mitochondrial NAD^+^ levels in cultured cells [36,115,116]. Intra-mitochondrial NAD^+^ has been described to be synthesized from NAM [32,117] or NMN via NMNAT3 [118]. In agreement with this hypothesis, a mitochondrial localized splice variant of NMNAT3 has been identified and ectopically expressed NMNAT3 shown to localize to the mitochondria [36]. Although one study suggested that NMNAT3 overexpression increased mitochondrial NAD^+^ [119], other studies reported that NMNAT3 was not required for mitochondrial NAD^+^ level maintenance [10,120]. Mitochondrial NAD^+^ levels vary in a circadian fashion and have been shown to directly influence fuel selection [121] and regulate cell survival under stress conditions [32]. Although cytoplasmic and mitochondrial NAD^+^ pools are indirectly connected via glycolysis and oxidative phosphorylation [122], very recent evidence has been presented using isotopic labeling of NR and NAR which suggest a direct NAD^+^ transport between these two intra-cellular spaces [123]. It is, therefore, possible that mitochondrial NAD^+^ comes from both, local synthesis and transport from the cytoplasm; each of these sources are likely to serve as the backup for the other, with both coordinately contributing to the tightly regulated and maintained NAD^+^ concentrations within mitochondria. 

### 3.2. Mitochondrial ADP-Ribosylation

**Figure 3:**
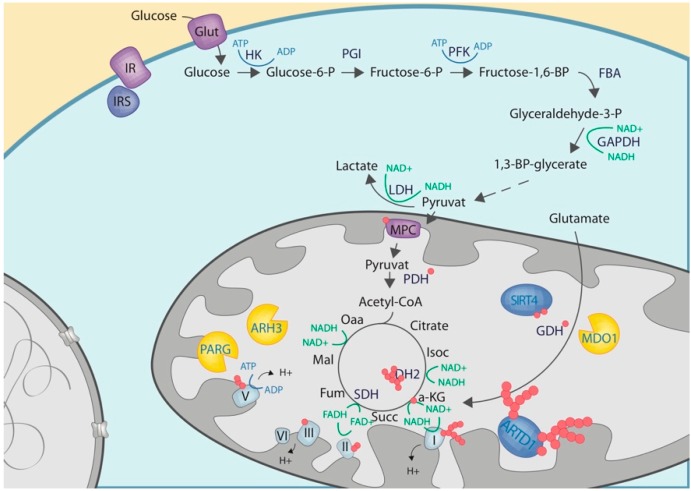
Schematic overview on the functional contribution of mitochondrial ARTs to carbohydrate metabolism. Metabolic pathways are shown as in Figure 1. ADP-ribosyltransferases are depicted in blue, while ADP-ribosylhydrolases are depicted in yellow.

High NAD^+^ concentrations within the mitochondria suggest that these organelles harbor ideal conditions for protein ADP-ribosylation to take place [29]. In fact, the concept of mitochondrial ADP-ribosylation was proposed over 30 years ago when a macromolecular enzymatic product of NAD^+^ was detected in mitochondria isolated from rat livers [117]. This, as well as several follow-up studies, further described ARTs and ADP-ribosylhydrolase (ARHs) activities in mitochondrial lysates [117,124,125]. Unfortunately, these studies failed to identify the corresponding enzymes that mediated these activities. Even the recent identification of the whole mitochondrial proteome did not result in the clear detection of any known ARTs in this organelle [126]. While the distribution of ARTs and ARHs in nucleus and cytoplasm has been quite well described, the existence and distribution of ADP-ribosylating enzymes in mitochondria remains thus controversial. Five enzymes able to regulate ADP-ribosylation have so far been associated with mitochondria: SIRT4 [94,127], ARTD1, PARG, ARH3 [92,128], and MacroD1 [93,129]. ART activity has, in fact, been detected in the mitochondrial matrix, as well as the inner- and outer mitochondrial membranes [130]. Due to the lack of clear evidence regarding mitochondrial localized ARTs and ARHs, this review briefly summarizes what is known about the five enzymes SIRT4, ARTD1, PARG, ARH3, and MacroD1 with respect to mitochondria:

*SIRT4:* In mammals, 7 SIRTs have been described, 3 of which (namely, SIRT3, 4, and 5) localize to the mitochondria (Table 2) [131,132,133]. Interestingly, SIRT4 has been described to not only hydrolyze protein bound acyl-groups, but to also possess ADP-ribosylation activity [94]. Indeed, SIRT4 was described to ADP-ribosylate and thus negatively regulate the activity of the glutamate dehydrogenase (GDH) in-vitro and in pancreatic β-cells, suggesting SIRT4 as an important regulator of insulin secretion [94,127]. GDH is, however, the only protein thus far described to be ADP-ribosylated by SIRT4 (Figure 3). Recent mass-spectrometry-based analyses identified various ADP-ribosylated mitochondria-localized proteins, thus further strengthening the idea that the mitochondria harboring several enzymes with ADP-ribosylating activities [75,76,134]. 

*ARTD1*: ARTD1s mitochondrial localization is the subject of controversial discussions in the field of ADP-ribosylation. This stems from the fact that ARTD1 does not contain a classical mitochondrial localization sequence and, thus, it has been proposed that its translocation is dependent on an association with mitofillin [135]. This association, however, would not explain how ARTD1 would be able to enter mitochondria. While some studies detected ARTD1 in mitochondria, mostly via western-blot analysis; other studies clearly only detect ARTD1 in the nucleus [135,136,137,138,139,140,141,142,143,144]. Due to the high abundance of ARTD1, western-blot analysis after cell fractionation (e.g., mitochondrial isolation) should to be taken with caution. Moreover, given that the proposed protein targets of mitochondria-localized ARTD1 (mtPARP1) mainly localize to the mitochondrial matrix, more thorough mitochondrial fractionations (including detergent lysis) are required to clarify if and where ARTD1 localizes within the mitochondria. Mitochondrial localization of ARTD1 has been proposed to regulate the mitochondrial energy homeostasis [92,139] (i.e., mitochondrial oxidative capacity) and mitochondrial DNA damage repair [145]. PARP inhibition or deletion of ARTD1 in different cell lines and systems has been shown to increase mitochondrial oxygen consumption rate [92]. Nevertheless, determining whether ARTD1 needs to localize to mitochondria for these effects requires further experimental investigation. In fact, since nuclear ARTD1 has also been demonstrated to affect whole cell NAD^+^ homeostasis [106], all metabolic phenotypes attributed to mitochondrial-localized ARTD1 might well be indirect consequences of the nuclear ARTD1 (see below, nuclear compartment). 

*PARG:* PARG is able to degrade poly-ADP-ribose [146]. Two shorter splice variants of PARG have been described to reside in the extra-nuclear space [92]. One of these shorter forms contains a predicted N-terminal mitochondrial localization sequence, but direct evidence demonstrating that this isoform of PARG localizes to the mitochondria remains missing. Nevertheless, overexpression studies targeting exogenous PARG (∆1-460) to mitochondria indicated that this PARG splice variant is inactive [92,128]. 

*ARH3:* ARH3 has recently been shown to hydrolyze both PARylation and MARylation [86,87]. While the majority of ARH3 localizes to the nucleus, a small fraction of the hydrolase has also been detected in mitochondria [86,87]. In contrast to PARG, overexpression of mitochondria-targeted ARH3 reduced mitochondrial protein ADP-ribosylation, thus suggesting that ARH3 might indeed be involved in mitochondrial ADPr metabolism [92,128]. Interestingly, while ARH3 could functionally be associated to metabolic diseases in human and in mice [147,148], direct evidence for endogenous mitochondrial ARH3, comparable to PARG, is still missing. 

*MacroD1:* MacroD1 has been shown to be active towards mono-ADP-ribosylated proteins, as well as toward free OAADPR, the by-product of SIRT-catalyzed de-acetylation [88,89,129]. In contrast to the four enzymes discussed above, MacroD1 has been clearly shown to predominantly localize to mitochondria [93,129]. Available transcriptome data from humans, mice, and rats [149] suggest that MacroD1 expression correlates with the energetic status of a given cell or tissue type. While the expression of MacroD1 is rather moderate in most tissues, tissues with a high energetic turnover, like the heart or skeletal muscle, express high levels of this hydrolase. In addition, several studies suggest that MacroD1 plays a role in the regulation of adipogenesis and insulin receptor signaling [150,151]. Upon knockdown of MacroD1 in adipocytes, an increase in glucose uptake and PPARγ expression on both mRNA and protein levels was observed; which suggests that it could negatively affect adipocyte differentiation. In addition, this knockdown was also shown to promote insulin signaling, which resulted in increased Akt phosphorylation [150]. In contrast, overexpression of MacroD1 increased ERK1/2- and Akt phosphorylation and insulin content in the pancreatic b-cell-derived MIN6 cell line [151]. While the precise mode of action of MacroD1 remains elusive, it is possible that MacroD1 influences the activity of Akt and insulin signaling in a cell and stimuli specific manner. In fact, MacroD1 has been shown to alter cell proliferation and apoptosis [152], and has been linked to the degree of differentiation and the invasiveness of various types of cancer [153]. The interesting associations of MacroD1 with cell metabolism and metabolism-related diseases make this enzyme an attractive target of further studies. 

The function and relevance of mitochondrial ADP-ribosylated proteins is currently under extensive debate. While some labs suggest mitochondrial ADP-ribosylation to be involved in the regulation of metabolic enzymes, others propose that the modification regulate mitochondrial DNA damage repair or oxidative stress responses [154]. Several recent proteomic studies identified various mitochondria-localized proteins to be ADP-ribosylated in lysates from both cells and mouse organs [75,76,134]. The amount of ADP-ribosylated mitochondrial proteins seemed especially pronounced in mitochondria-rich tissues with high metabolic activities such as heart, skeletal muscle, and brown adipose tissue. Nevertheless, detection of endogenous mitochondrial ADP-ribosylation via other methods has thus far proven challenging. Various ADP-ribosylated mitochondrial proteins are implicated in many different biological processes, including mtDNA repair, protein-, metabolite- and ion transport, protein folding and synthesis, and mitochondrial metabolism. The function of ADP-ribosylation in mtDNA repair, has however thoroughly been addressed in previous reviews [154]. As this review focuses mainly on carbohydrate metabolism, proteins involved in non-metabolic processes will not further be discussed.

### 3.3. Crosstalk with Proteins or Enzymes of the Carbohydrate Metabolisms

*Complex I, II, III, and the ATP-synthase:* A prominent cluster of mitochondrial ADP-ribosylated proteins is involved in the mitochondrial respiratory chain. Indeed, complex I, II, III, and the ATP-synthase have been shown to be ADP-ribosylated and harbor various potential ADPr acceptor sites [75] (Figure 3). Several studies, including an in-vivo ischemia reperfusion (or traumatic brain injury model) and in vitro activation of the nitric-oxide synthase, provided evidence that the induction of ADP-ribosylation dampens mitochondrial oxidative phosphorylation [136,137,139,155]. While some of the studies directly showed different sub-units of different respiratory chain complexes to be ADP-ribosylated upon induction of the respective stress, other studies only observed an increase in (mitochondrial) ADP-ribosylation in general. 

*Malate dehydrogenase (MDH), pyruvate dehydrogenase (PDH) and glutamate dehydrogenase (GDH):* In addition to the respiratory chain components, other mitochondrial metabolic enzymes involved in carbohydrate metabolism, such as MDH, PDH or GDH were also found to be ADP-ribosylated [75,94,134]. While the functional contribution of ADP-ribosylation to MDH or PDH activities has not yet been experimentally addressed, ADP-ribosylation of the GDH by SIRT4 has been proposed to dampen its activity (see above) [94]. 

## 4. Crosstalk between Nuclear ADP-Ribosylation and the Carbohydrate Metabolism

### 4.1. Nuclear NAD Biosynthesis

The cytoplasmic NAD^+^ pool serves as a central hub that connects and regulates other NAD^+^ pools (Figure 1). Cytosolic and nuclear NAD^+^ pools are closely connected as indicated by studies using the small molecule NAMPT inhibitor FK866, which demonstrated comparable nuclear and cytoplasmic reductions in NAD^+^ following treatment [13]. It is also expected that the NAD^+^ precursors, NAM and NMN, are shared between nucleus and cytosol as they are able to freely diffuse through the nuclear pore complex. The final step, converting NMN to NAD^+^, is believed to be locally catalyzed. While cytoplasmic NAD^+^ is mainly synthesized by NMNAT2, which localizes to the Golgi apparatus [35,156], nuclear NAD^+^ is thought to be mainly synthesized by nuclear NMNAT1, the most ubiquitously expressed NMNAT isoform [157,158]. Recent studies found that depleting NMNAT2 significantly reduced cytoplasmic NAD^+^ but minimally affected nuclear NAD^+^. Furthermore, nuclear NAD^+^ could not completely replenish cytoplasmic NAD^+^ following NMNAT2 depletion [13]. Additionally, NMNAT1 has been shown to be recruited to gene promoters targeted by ARTD1, to locally generate NAD^+^ and support the catalytic activity of ARTD1 [69,158]. These observations suggest that while nuclear and cytoplasmic NAD^+^ pools are interconnected, local regulation by NMNAT1 and NMNAT2, respectively, still plays an important role in NAD^+^ homeostasis. This compartment-specific local regulation of NAD^+^ biosynthesis seems highly important for controlling the enzymatic activities of ARTs and SIRTs. 

### 4.2. Nuclear ADP-riboslyation 

The nuclear role of NAD^+^ is mainly centered on ARTD-mediated ADP-ribosylation and SIRT- mediated deacetylation. As ARTDs and SIRTs are NAD^+^-consuming enzymes, their activities require quasi constant replenishment of nuclear NAD^+^, mainly through nuclear NMNAT1-mediated NAD^+^ synthesis [9]. Nuclear ARTDs can dampen the activity of SIRTs either directly via ADP-ribosylation or indirectly via substrate competition. Given that nuclear ARTs, especially ARTD1, are able to consume tremendous amounts of NAD^+^ upon hyperactivation, they can reduce the substrates/co-factors (NAD^+^, ATP) of many metabolic enzymes. Three of the known ARTDs localize predominantly to the nucleus (ARTD1, ARTD2 and ARTD3), while others shuttle between the cytoplasm and nucleus (ARTD4-6, ARTD8-12, ARTD14) [159]. ARTD1 is the most well-characterized member of the ARTD family and is best-known for its involvement in genotoxic stress responses and as a cleaved marker during apoptosis [160]. ARTD1 however also regulates transcription by different mechanisms including the binding and/or modification of transcription factors (see below), transcription co-factors or histones and thus influencing highly organized chromatin structures [161,162,163,164,165]. Of the seven mammalian SIRTs, SIRT1, 6 and 7 have all been shown to localized to the nucleus [57,166]. SIRT6 has been described to be an ADP-ribosyltransferase [91] that directly modifies ARTD1 and BAF170 [167,168], the latter of which is a subunit of BAF chromatin remodeling complex required for activation of a subset of NRF2 responsive genes following oxidative stress [167]. The extensive network of ARTD and SIRT proteins and their regulatory targets indicates the profound impact that nuclear NAD^+^ can have on various cellular processes [29]. A direct link between the enzymatic activities of nuclear ARHs and the metabolic state of the cell remains to be explored.

### 4.3. Crosstalk with Proteins Regulating the Expression of Enzymes Involved in the Carbohydrate Metabolisms

As most enzymes involved in carbohydrate metabolism reside in either the cytoplasm or the mitochondria, nuclear ARTDs have a limited ability to influence their activity directly via ADP-ribosylation. As discussed above, the exception is GAPDH, which is believed to shuttle between the cytoplasm and nucleus in response to metabolic stimulation. Nevertheless, nuclear ARTDs may still indirectly affect various metabolic enzymes; indeed, it is possible that they regulate the expression of genes involved in metabolic processes, potentially via transcription factor ADP-ribosylation (see above). 

**Figure 4:**
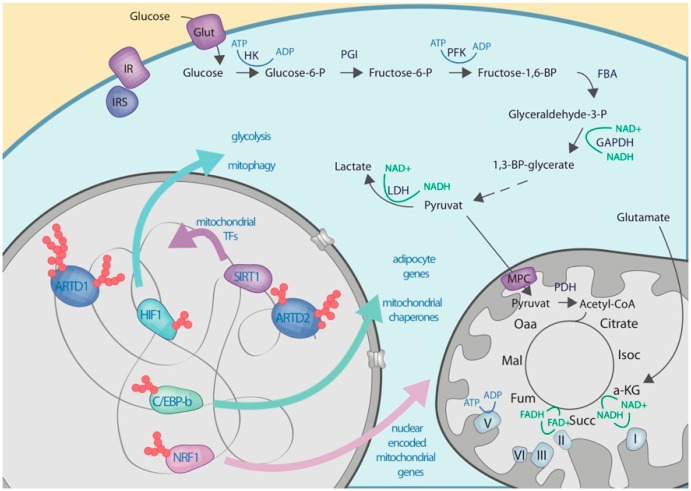
Schematic overview on the functional contribution of nuclear ARTs to carbohydrate metabolism. Metabolic pathways are shown as in Figure 1.

*C/EBP-b:* C/EBP-b is an important transcription factor involved in various physiological processes including embryonic development, immune responses, hematopoiesis, adipogenesis, and glucose metabolism [169,170,171]. C/EBP-b is a direct target of ARTD1-mediated poly-ADP-ribosylation during adipocyte differentiation [69] (Figure 4). In fact, ADP-ribosylation of C/EBP-b during adipogenesis initiation results in its eviction from the DNA. In order to allow C/EBP-b to bind to and activate its target genes during the initial phase of adipogenesis, the activity of ARTD1 had to be dampened via reduction of local NAD^+^ [69]. Adipogenic signaling rapidly induces cytoplasmic NMNAT-2, which competed with nuclear NMNAT-1 for the NMN leading to a reduction in nuclear NAD^+^ levels. The whole process, including the changes in cytoplasmic and nuclear NAD^+^ levels was dependent on glucose-uptake. Interestingly, cells would still differentiate in a glucose-free medium if ARTD1 was knocked down, pointing towards an interplay between ARTD1 activity, NAD^+^ availability, and glucose metabolism. 

*NRF1:* Nuclear respiratory factor 1 (NRF1) is a transcription factor that regulates cell growth and metabolism by activating genes involved in mitochondrial biogenesis and function [172]. Together with NRF2, NRF1 mediates nuclear- and the mitochondrial genome coordination by regulating the expression of a cluster of nuclear DNA-encoded genes that localize to the mitochondria [172]. ARTD1 has been shown to form a complex with NRF1 in an ADPr-independent manner facilitating the regulation of its target genes [173] (Figure 4). Although modification is not required for the binding of NRF1 to ARTD1, its enzymatic activity seems to be important for NRF1 transcriptional activity. While the functional contribution of NRF1 in the regulation of mitochondrial dynamics and function is well described, the precise contribution of ARTD1 remains to be investigated. Nonetheless, as ARTD1 activity does affect mitochondrial function and cell metabolism, it is interesting to speculate that ARTD1 interacts with NRF1 in an NAD^+^-dependent manner, and that this interaction transmits information about the metabolic status (i.e., NAD^+^ concentration/availability) of the cell between nucleus and mitochondria.

*HIF1:* Hypoxia-inducible factor (HIF) 1 is the key transcription factor that regulates gene expression in response to hypoxia [174]. The family of human HIF transcription factors is composed of six members, all of which respond to changes in oxygen availability [174]. Upon oxygen deprivation, HIF1 is stabilized and regulates the adaptation of the cells toward hypoxia on the transcriptional level. Many genes involved in this adaptation are metabolic, and this is because hypoxic conditions force cells to rely on glycolysis rather than oxidative phosphorylation [174]. Several studies have demonstrated that ARTD1 forms a complex with HIF1 and acts as a transcriptional co-activator for HIF1 target genes [175,176] (Figure 4). Although HIF1 does not seem to be a direct target of ARTD1, this co-activation has been shown to be ADP-ribosylation dependent, as treatment with PARP inhibitors impaired HIF1 target gene activation [175,176]. 

*SIRT1:* SIRT1 is a nuclear de-acetylase involved in the transcriptional regulation of various genes [177]. Expression and activation of SIRT1 is generally positively associated with whole cell NAD^+^ levels and longevity [177,178]. *ARTD2* deficiency resulted in an increase in SIRT1 on mRNA and protein level, suggesting that ARTD2 negatively regulates the activity of SIRT1 in cell lines and mice [179,180,181,182] (Figure 4). In line with this, *ARTD2*-deficient cells and mice showed higher expression levels of SIRT1 regulated genes, such as PGC-1a, and displayed increased mitochondrial biogenesis and function [179]. Further, *ARTD2*-deficient animals were protected against diet-induced obesity [179]. Inversely, overexpression of *ARTD2* decreased SIRT1 levels and mitochondrial biogenesis [180]. The effect of ARTD2 on SIRT1 was found to be NAD^+^ independent, thus excluding a NAD^+^ substrate competition.

*KAP1:* KRAB-associated protein 1 (KAP1) is an important regulator of chromatin organization involved in numerous processes including embryogenesis, development, cell cycle progression, and apoptosis [183]. KAP1 also plays a important role in the repression of LINE1 transposons that depends on SIRT6-mediated ADP-ribosylation of KAP1 [184]. The association of SIRT6 to LINE1 loci and the modification of KAP1 declines with age. However, the mechanism how KAP1ADP-ribosylation is regulating cell metabolism remains to be investigated.

## 5. Intercompartmental NAD^+^ Cross-Talks after Genotoxic Stress

Many enzymes involved in glycolysis and oxidative phosphorylation require NAD^+^ or ATP as co-factors for their reactions (Figure 1, Figure 2 and Figure 3). Consequently, a decrease in cytoplasmic and/or mitochondrial NAD^+^ levels, and subsequently a decrease in whole cell ATP levels, blocks various anabolic and catabolic reactions due to co-factor deficiencies. Studies using either PARP-inhibitors (PARPi) or *ARTD1*-deficient cells revealed an anti-correlation between ARTD1 activity and intracellular NAD^+^ levels under basal conditions. Thus, implying that ARTD1 also consumes NAD^+^ under physiological conditions [9]. Hyperactivation of ARTD1, as observed following severe genotoxic stress, has also been associated with a substrate competition-mediated decrease in SIRT1 activity [185]. While some studies mainly observed a considerable drop in nuclear and cytoplasmic NAD^+^ levels following genotoxic stress, other studies have also found that mitochondrial NAD^+^ levels drop under these conditions [68,72,106,186]. Several studies have further associated genotoxic stress induced ARTD1 activation with a drop in ATP levels and glycolysis [68,72,186]. It is important to note that while some studies observed mainly a decrease in glycolysis, other studies also observed reduced mitochondrial oxidative phosphorylation [106]. Both phenotypes could not only be rescued via ARTD1 inhibition/knockout, but also via NAD^+^-supplementation [68,72,186]. The fact that PARP inhibitor increased NAD^+^ levels in mice, improved mitochondrial function, and protected against the effects of a high fat diet [187,188], provides strong evidence for a intercompartmental cross-talk. Thus, two main hypotheses exist that could explain how hyper-activation of ARTD1 affects cell metabolism: (i) studies describing a drop in glycolysis suggest that severe genotoxic stress results in a metabolic switch from glycolysis to oxidative phosphorylation, making cells dependent on mitochondria for energy/ATP production, or (ii) studies demonstrating a reduction in both the glycolytic and TCA capacity point towards a general reduction in cell metabolism following ARTD1 activation and subsequent NAD^+^ depletion. Along with the first possibility, supplementation with non-glucose carbon sources, such as pyruvate or glutamate to directly fuel the TCA cycle, rescued astrocyte cell death following MNNG treatment [68]. In fact, the two hypotheses presented here are not mutually exclusive as the reduced cellular metabolic capacities could be a direct function of the degree of ARTD1 activation. While mild activation of ARTD1 might predominantly result in the depletion of cytoplasmic and nuclear NAD^+^ pools, more pronounced activation might also considerably deplete mitochondrial NAD^+^. As a consequence, in the first instance, cells would mainly experience a decrease in glycolysis as they would run out of cytoplasmic NAD^+^, which is required for the conversion of glucose-3-phosphate to 1,3-bisphosphoglycerate. Following a more pronounced drop in NAD^+^ levels, cells would experience further reductions in TCA capacity, as well as in oxidative phosphorylation. Pyruvate and glutamate could reduce the severity of the phenotype in both cases, as both metabolites are not exclusively metabolized within the mitochondria, but can also be utilized by enzymes in the cytoplasm [78].

Whether mitochondria, with their high NAD^+^ concentrations, also contribute to NAD^+^-regulated processes in other cellular compartments requires additional investigation. However, increased mitochondrial NAD^+^ promoted cell survival during genotoxic stress, which was regulated by the mitochondrial SIRT3 and SIRT4 [32]. These data show that mitochondrial NAD^+^ is a major determinant of apoptosis and shed new light on the influence of diet on organ physiology and disease.

## Figures and Tables

**Table 1:** Overview of ADP-ribosylated proteins involved in glucose metabolism with assigned ADP-ribose amino acid acceptor site and their function.

Protein	Modification Side	Function	Function Affected by ADPR	Localization	References
ATP5A1	E508, K506 (m) S184, S513 (h)	ATP synthesis	N/A	m	[75,76]
ATP5B	E98, R121 (m)	ATP synthesis	N/A	m	[75]
ATP5F1	H164, S226 (h)	ATP synthesis	N/A	m	[76]
ATP5F1B	S415, R458, H477 (h)	ATP synthesis	N/A	m	[76]
ATP5F1C	S195 (h)	ATP synthesis	N/A	m	[76]
ATP50	R29, S126 (m) S163, S166 (h)	ATP synthesis	N/A	m	[75,76]
C/EBP-b	E135, K133, E139 (m) S141, S257 (h)	Transcription factor, gene expression	Yes (activity dampened)	n	[76,77]
COX4I1	H51 (h)	Electron transport chain	N/A	m	[76]
CYCS	S34 (h)	Electron transport chain	N/A	m	[76]
GAPDH	R198, R232 (m) R80, S122, S192, R197, S210 (h)	Glycolysis	N/A	c, n	[75,76]
MDH1	S241 (h)	Carbohydrate metabolism	N/A	m	[76]
MDH2	S261, S246 (m) S317 (h)	Carbohydrate metabolism	N/A	m	[75,76]
NDUFB1	R49 (m)	Electron transport	N/A	m	[75]
NDUFB5	R93 (m)	Electron transport	N/A	m	[75]
NDUFAF7	R415 (m)	Electron transport	N/A	m	[75]
NDUFC2	R120 (m)	Electron transport	N/A	m	[75]
NDUFAB1	S99 (h)	Electron transport	N/A	m	[76]
NDUFV1	R449 (m)	Electron transport	N/A	m	[75]
PDPR	R866 (m)	Carbohydrate metabolism	N/A	m	[75]
PDHA1	R304 (m)	Carbohydrate metabolism	N/A	m	[75]
PDHX	S131 (m)	Carbohydrate metabolism	N/A	m	[75]
SDHA	S505, H522 (h)	Electron transport	N/A	m	[76]
UQCRC1	S221 (h)	Electron transport	N/A	m	[76]
UQCRC2	R241 (m) S221 (h)	Electron transport	N/A	m	[75,76]
UQCRFS1	E95 (m)	Electron transport	N/A	m	[75]

Abbreviations: R: arginine, S: serine, K: lysine, H: histidine, E: glutamic acid, c: cytoplasm, m: mitochondria, n: nucleus, (m): mouse, (h): human, N/A: not available.

**Table 2:** Overview of the localization of enzymes involved in NAD^+^ synthesis and conversion.

**Nucleus**				
**Family**	**Name**	**NAD^+^ Metabolism**	**ART/ARH Activity**	**References**
ARTs	ARTD1	Consumption	Poly (branching)	[79,80,81]
	ARTD2	Consumption	Poly (branching)	[79,80,81]
	ARTD3	Consumption	Mono	[79,80,81]
	ARTD4	Consumption	Mono	[79,80,81]
	ARTD5	Consumption	Poly/Oligo	[79,80,81]
	ARTD6	Consumption	Poly/Oligo	[79,80,81]
	ARTD8	Consumption	Mono	[79,80,81]
	ARTD9	Consumption	Inactive/Mono	[79,80,82]
	ARTD10	Consumption	Mono	[79,80,81]
	ARTD11	Consumption	Mono	[79,80,81]
	ARTD14	Consumption	Mono	[79,80,81]
ARHs	PARG		Poly	[83,84]
	ARH3		Poly/Mono	[85,86,87]
	TARG		Mono	[88,89,90]
SIRTs	SIRT1	Consumption	N/A	[39]
	SIRT2	Consumption	N/A	[39]
	SIRT6	Consumption	Mono	[39,91]
	SIRT7	Consumption	N/A	[39]
NAMPT	NAMPT	Synthesis	N/A	[2]
NMNAT	NMNAT1	Synthesis	N/A	[2]
**Cytoplasm**				
**Family**	**Name**	**NAD^+^ Metabolism**	**ART/ARH Activity**	**References**
ARTs	ARTD2	Consumption	Poly (branching)	[79,80,81]
	ARTD3	Consumption	Mono	[79,80,81]
	ARTD4	Consumption	Mono	[79,80,81]
	ARTD5	Consumption	Poly/Oligo	[79,80,81]
	ARTD6	Consumption	Poly/Oligo	[79,80,81]
	ARTD7	Consumption	Mono	[79,80,81]
	ARTD8	Consumption	Mono	[79,80,81]
	ARTD9	Consumption	Inactive/Mono	[79,80,82]
	ARTD10	Consumption	Mono	[79,80,81]
	ARTD11	Consumption	Mono	[79,80,81]
	ARTD12	Consumption	Mono	[79,80,81]
	ARTD13	Consumption	Inactive	[79,80,81]
	ARTD14	Consumption	Mono	[79,80,81]
	ARTD15	Consumption	Mono	[79,80,81]
	ARTD16	Consumption	Mono	[79,80,81]
	ARTD17	Consumption	Mono	[79,80,81]
	ARTD18	Consumption	Mono	[79,80,81]
ARHs	PARG		Poly	[83,84]
	ARH1		Mono	[85]
	ARH2		Inactive	[85]
	MacroD2		Mono	[88,89,90]
SIRTs	SIRT1	Consumption	N/A	[39]
	SIRT2	Consumption	N/A	[39]
NAMPT	NAMPT	Synthesis	N/A	[2]
NMNAT	NMNAT2	Synthesis	N/A	[2]
**Mitochondria**				
**Family**	**Name**	**NAD^+^ Metabolism**	**ART/ARH Activity**	**References**
ARTs	ARTD1	Consumption	Poly (branching)	[79,80]
ARHs	PARG		Poly	[83,84,92]
	ARH3		Poly/Mono	[85,86,87]
	MacroD1		Mono	[88,89,90,93]
SIRTs	SIRT3	Consumption	N/A	[39]
	SIRT4	Consumption	Mono	[39,94]
	SIRT5	Consumption	N/A	[39]
NAMPT	NAMPT	Synthesis	N/A	[2]
NMNAT	NMNAT3	Synthesis	N/A	[2]

Abbreviations: ART: ADP-ribosyltransferase, ARH: ADP-ribosylhydrolase, SIRT: Sirtuin, NAMPT: Nicotinamide phosphoribosyltransferase, NMNAT: Nicotinamide mononucleotide adenylyltransferase, N/A: Not available
