# Erratum: Hopp, A.K., et al. Regulation of Glucose Metabolism by NAD^+^ and ADP-Ribosylation. *Cells* 2019, *8*, 890

**DOI:** 10.3390/cells8111371

**Published:** 2019-10-31

**Authors:** Ann-Katrin Hopp, Patrick Grüter, Michael O. Hottiger

**Affiliations:** 1 Department of Molecular Mechanisms of Disease (DMMD), University of Zurich, CH-8057 Zurich, Switzerland; 2 Molecular Life Science Ph.D. Program, Life Science Zurich Graduate School, CH-8057 Zurich, Switzerland

Change in References list order.

The authors wish to make the following corrections to this review [1], since in the published version the reference citation in the main text does not correlate properly to the correct reference list numbers. The order of references from ref [75] to ref [188] should be as listed below. The authors would like to apologize for any inconvenience caused to the readers by these changes. The change does not affect statements or conclusions of the review.

75. Leutert, M.; Menzel, S.; Braren, R.; Rissiek, B.; Hopp, A.K.; Nowak, K.; Bisceglie, L.; Gehrig, P.; Li, H.; Zolkiewska, A.; et al. Proteomic Characterization of the Heart and Skeletal Muscle Reveals Widespread Arginine ADP-Ribosylation by the ARTC1 Ectoenzyme. *Cell Rep.* **2018**, doi:10.1016/j.celrep.2018.07.048.
76. Larsen, S.C.; Hendriks, I.A.; Lyon, D.; Jensen, L.J.; Nielsen, M.L. Systems-wide Analysis of Serine ADP-Ribosylation Reveals Widespread Occurrence and Site-Specific Overlap with Phosphorylation. *Cell Rep.* **2018**, doi:10.1016/j.celrep.2018.07.083.
77. Luo, X.; Ryu, K.W.; Kim, D.S.; Nandu, T.; Medina, C.J.; Gupte, R.; Gibson, B.A.; Soccio, R.E.; Yu, Y.; Gupta, R.K.; et al. PARP-1 Controls the Adipogenic Transcriptional Program by PARylating C/EBPβ and Modulating Its Transcriptional Activity. *Mol. Cell* **2017**, doi:10.1016/j.molcel.2016.11.015.
78. Akram, M. Citric Acid Cycle and Role of its Intermediates in Metabolism. *Cell Biochem. Biophys.* **2014**, doi:10.1007/s12013-013-9750-1.
79. Vyas, S.; Matic, I.; Uchima, L.; Rood, J.; Zaja, R.; Hay, R.T.; Ahel, I.; Chang, P. Family-wide analysis of poly(ADP-ribose) polymerase activity. *Nat. Commun.* **2014**, doi:10.1038/ncomms5426.
80. Vyas, S.; Chesarone-Cataldo, M.; Todorova, T.; Huang, Y.H.; Chang, P. A systematic analysis of the PARP protein family identifies new functions critical for cell physiology. *Nat. Commun.* **2013**, doi:10.1038/ncomms3240.
81. Verheugd, P.; Bütepage, M.; Eckei, L.; Lüscher, B. Players in ADP-ribosylation: Readers and Erasers. *Curr. Protein Pept. Sci.* **2016**, *17*, 654–667.
82. Yang, C.S.; Jividen, K.; Spencer, A.; Dworak, N.; Ni, L.; Oostdyk, L.T.; Chatterjee, M.; Kuśmider, B.; Reon, B.; Parlak, M.; et al. Ubiquitin Modification by the E3 Ligase/ADP-Ribosyltransferase Dtx3L/Parp9. *Mol. Cell* **2017**, doi:10.1016/j.molcel.2017.04.028.
83. Slade, D.; Dunstan, M.S.; Barkauskaite, E.; Weston, R.; Lafite, P.; Dixon, N.; Ahel, M.; Leys, D.; Ahel, I. The structure and catalytic mechanism of a poly(ADP-ribose) glycohydrolase. *Nature* **2011**, doi:10.1038/nature10404.
84. Ohashi, S.; Kanai, M.; Hanai, S.; Uchiumi, F.; Maruta, H.; Tanuma, S.I.; Miwa, M. Subcellular localization of poly(ADP-ribose) glycohydrolase in mammalian cells. *Biochem. Biophys. Res. Commun.* **2003**, doi:10.1016/S0006-291X(03)01272-5.
85. Mashimo, M.; Kato, J.; Moss, J. Structure and function of the ARH family of ADP-ribosyl-acceptor hydrolases. *DNA Repair (Amst).* **2014**, doi: 10.1016/j.dnarep.2014.03.005.
86. Abplanalp, J.; Leutert, M.; Frugier, E.; Nowak, K.; Feurer, R.; Kato, J.; Kistemaker, H.V.A.; Filippov, D.V.; Moss, J.; Caflisch, A.; et al. Proteomic analyses identify ARH3 as a serine mono-ADP-ribosylhydrolase. *Nat. Commun.* **2017**, doi:10.1038/s41467-017-02253-1.
87. Fontana, P.; Bonfiglio, J.J.; Palazzo, L.; Bartlett, E.; Matic, I.; Ahel, I. Serine ADP-ribosylation reversal by the hydrolase ARH3. *Elife* **2017**, doi:10.7554/eLife.28533.
88. Rosenthal, F.; Feijs, K.L.H.; Frugier, E.; Bonalli, M.; Forst, A.H.; Imhof, R.; Winkler, H.C.; Fischer, D.; Caflisch, A.; Hassa, P.O.; et al. Macrodomain-containing proteins are new mono-ADP-ribosylhydrolases. *Nat. Struct. Mol. Biol.* **2013**, doi:10.1038/nsmb.2521.
89. Jankevicius, G.; Hassler, M.; Golia, B.; Rybin, V.; Zacharias, M.; Timinszky, G.; Ladurner, A.G. A family of macrodomain proteins reverses cellular mono-ADP-ribosylation. *Nat. Struct. Mol. Biol.* **2013**, doi:10.1038/nsmb.2523.
90. Feijs, K.L.H.; Forst, A.H.; Verheugd, P.; Lüscher, B. Macrodomain-containing proteins: Regulating new intracellular functions of mono(ADP-ribosyl)ation. *Nat. Rev. Mol. Cell Biol.* **2013**, doi:10.1038/nrm3601.
91. Liszt, G.; Ford, E.; Kurtev, M.; Guarente, L. Mouse Sir2 homolog SIRT6 is a nuclear ADP-ribosyltransferase. *J. Biol. Chem.* **2005**, doi:10.1074/jbc.M413296200.
92. Niere, M.; Kernstock, S.; Koch-Nolte, F.; Ziegler, M. Functional Localization of Two Poly(ADP-Ribose)-Degrading Enzymes to the Mitochondrial Matrix. *Mol. Cell. Biol.* **2008**, doi:10.1128/MCB.01766-07.
93. Agnew, T.; Munnur, D.; Crawford, K.; Palazzo, L.; Mikoc, A.; Ahel, I. MacroD1 is a promiscuous ADP-ribosyl hydrolase localized to mitochondria. *Front. Microbiol.* **2018**, doi:10.3389/fmicb.2018.00020.
94. Haigis, M.C.; Mostoslavsky, R.; Haigis, K.M.; Fahie, K.; Christodoulou, D.C.; Murphy, A.J.J.; Valenzuela, D.M.; Yancopoulos, G.D.; Karow, M.; Blander, G.; et al. SIRT4 Inhibits Glutamate Dehydrogenase and Opposes the Effects of Calorie Restriction in Pancreatic β Cells. *Cell* **2006**, doi:10.1016/j.cell.2006.06.057.
95. Grimaldi, G.; Corda, D. ADP-ribosylation and intracellular traffic: an emerging role for PARP enzymes. *Biochem. Soc. Trans.* **2019**, doi:10.1042/BST20180416.
96. Catara, G.; Grimaldi, G.; Schembri, L.; Spano, D.; Turacchio, G.; Lo Monte, M.; Beccari, A.R.; Valente, C.; Corda, D. PARP1-produced poly-ADP-ribose causes the PARP12 translocation to stress granules and impairment of Golgi complex functions. *Sci. Rep.* **2017**, doi:10.1038/s41598-017-14156-8.
97. Jwa, M.; Chang, P. PARP16 is a tail-anchored endoplasmic reticulum protein required for the PERK-and IRE1α-mediated unfolded protein response. *Nat. Cell Biol.* **2012**, doi:10.1038/ncb2593.
98. Di Paola, S.; Micaroni, M.; Di Tullio, G.; Buccione, R.; Di Girolamo, M. PARP16/ARTD15 is a novel endoplasmic-reticulum-associated mono-ADP-ribosyltransferase that interacts with, and modifies karyopherin-ß1. *PLoS ONE* **2012**, doi:10.1371/journal.pone.0037352.
99. Yeh, T.Y.J.; Beiswenger, K.K.; Li, P.; Bolin, K.E.; Lee, R.M.; Tsao, T.S.; Murphy, A.N.; Hevener, A.L.; Chi, N.W. Hypermetabolism, hyperphagia, and reduced adiposity in tankyrase-deficient mice. *Diabetes* **2009**, doi:10.2337/db08-1781.
100. Feijs, K.L.; Kleine, H.; Braczynski, A.; Forst, A.H.; Herzog, N.; Verheugd, P.; Linzen, U.; Kremmer, E.; Lüscher, B. ARTD10 substrate identification on protein microarrays: Regulation of GSK3β by mono-ADP-ribosylation. *Cell Commun. Signal.* **2013**, doi:10.1186/1478-811X-11-5.
101. Lüscher, B.; Bütepage, M.; Eckei, L.; Krieg, S.; Verheugd, P.; Shilton, B.H. ADP-Ribosylation, a Multifaceted Posttranslational Modification Involved in the Control of Cell Physiology in Health and Disease. *Chem. Rev.* **2018**, doi:10.1021/acs.chemrev.7b00122.
102. Mueckler, M.; Thorens, B. The SLC2 (GLUT) family of membrane transporters. *Mol. Aspects Med.* **2013**, doi:10.1016/j.mam.2012.07.001.
103. Huang, S.; Czech, M.P. The GLUT4 Glucose Transporter. *Cell Metab.* **2007**, doi:10.1016/j.cmet.2007.03.006.
104. Su, Z.; Deshpande, V.; James, D.E.; Stöckli, J. Tankyrase modulates insulin sensitivity in skeletal muscle cells by regulating the stability of GLUT4 vesicle proteins. *J. Biol. Chem.* **2018**, doi:10.1074/jbc.RA117.001058.
105. Robey, R.B.; Hay, N. Mitochondrial hexokinases, novel mediators of the antiapoptotic effects of growth factors and Akt. *Oncogene* **2006**, doi:10.1038/sj.onc.1209595.
106. Fouquerel, E.; Goellner, E.M.; Yu, Z.; Gagné, J.P.; de Moura, M.B.; Feinstein, T.; Wheeler, D.; Redpath, P.; Li, J.; Romero, G.; et al. ARTD1/PARP1 negatively regulates glycolysis by inhibiting hexokinase 1 independent of NAD + depletion. *Cell Rep.* **2014**, doi:10.1016/j.celrep.2014.08.036.
107. Lochhead, P.A.; Coghlan, M.; Rice, S.Q.J.; Sutherland, C. Inhibition of GSK-3 selectively reduces glucose-6-phosphatase and phosphoenolpyruvate carboxykinase gene expression. *Diabetes* **2001**, doi:10.2337/diabetes.50.5.937.
108. Liberman, Z.; Eldar-Finkelman, H. Serine 332 phosphorylation of insulin receptor substrate-1 by glycogen synthase kinase-3 attenuates insulin signaling. *J. Biol. Chem.* **2005**, doi:10.1074/jbc.M410610200.
109. Márton, J.; Fodor, T.; Nagy, L.; Vida, A.; Kis, G.; Brunyánszki, A.; Antal, M.; Lüscher, B.; Bai, P. PARP10 (ARTD10) modulates mitochondrial function. *PLoS ONE* **2018**, doi:10.1371/journal.pone.0187789.
110. Nicholls, C.; Li, H.; Liu, J.P. GAPDH: A common enzyme with uncommon functions. *Clin. Exp. Pharmacol. Physiol.* **2012**, doi:10.1111/j.1440-1681.2011.05599.x.
111. Sirover, M.A. Pleiotropic effects of moonlighting glyceraldehyde-3-phosphate dehydrogenase (GAPDH) in cancer progression, invasiveness, and metastases. *Cancer Metastasis Rev.* **2018**, doi:10.1007/s10555-018-9764-7.
112. Kots, A.Y.; Sergienko, E.A.; Bulargina, T.V.; Severin, E.S. Glyceraldehyde-3-phosphate activates auto-ADP-ribosylation of glyceraldehyde-3-phosphate dehydrogenase. *FEBS Lett.* **1993**, doi:10.1016/0014-5793(93)81526-6.
113. Du, X.; Matsumura, T.; Edelstein, D.; Rossetti, L.; Zsengellér, Z.; Szabó, C.; Brownlee, M. Inhibition of GAPDH activity by poly(ADP-ribose) polymerase activates three major pathways of hyperglycemic damage in endothelial cells. *J. Clin. Invest.* **2003**, doi:10.1172/JCI18127.
114. Mayo, E.; Fabrizio, G.; Scarpa, E.; Stilla, A.; Dani, N.; Chiacchiera, F.; Kleine, H.; Attanasio, F.; Lüscher, B.; Di Girolamo, M. ARTD10/PARP10 Induces ADP-Ribosylation of GAPDH and Recruits GAPDH into Cytosolic Membrane-Free Cell Bodies When Overexpressed in Mammalian Cells. *Challenges* **2018**, doi:10.3390/challe9010022.
115. Cantó, C.; Houtkooper, R.H.; Pirinen, E.; Youn, D.Y.; Oosterveer, M.H.; Cen, Y.; Fernandez-Marcos, P.J.; Yamamoto, H.; Andreux, P.A.; Cettour-Rose, P.; et al. The NAD+ precursor nicotinamide riboside enhances oxidative metabolism and protects against high-fat diet-induced obesity. *Cell Metab.* **2012**, doi:10.1016/j.cmet.2012.04.022.
116. Pittelli, M.; Felici, R.; Pitozzi, V.; Giovannelli, L.; Bigagli, E.; Cialdai, F.; Romano, G.; Moroni, F.; Chiarugi, A. Pharmacological Effects of Exogenous NAD on Mitochondrial Bioenergetics, DNA Repair, and Apoptosis. *Mol. Pharmacol.* **2011**, doi:10.1124/mol.111.073916.
117. Kun, E.; Zimber, P.H.; Chang, A.C.; Puschendorf, B.; Grunicke, H. Macromolecular enzymatic product of NAD+ in liver mitochondria. *Proc. Natl. Acad. Sci.* **1975**, doi:10.1073/pnas.72.4.1436.
118. Nikiforov, A.; Dölle, C.; Niere, M.; Ziegler, M. Pathways and subcellular compartmentation of NAD biosynthesis in human cells: From entry of extracellular precursors to mitochondrial NAD generation. *J. Biol. Chem.* **2011**, doi:10.1074/jbc.M110.213298.
119. Son, M.J.; Kwon, Y.; Son, T.; Cho, Y.S. Restoration of Mitochondrial NAD+ Levels Delays Stem Cell Senescence and Facilitates Reprogramming of Aged Somatic Cells. *Stem Cells* **2016**, doi:10.1002/stem.2460.
120. Yamamoto, M.; Hikosaka, K.; Mahmood, A.; Tobe, K.; Shojaku, H.; Inohara, H.; Nakagawa, T. Nmnat3 is dispensable in mitochondrial NAD level maintenance in vivo. *PLoS ONE* **2016**, doi:10.1371/journal.pone.0147037.
121. Peek, C.B.; Affinati, A.H.; Ramsey, K.M.; Kuo, H.Y.; Yu, W.; Sena, L.A.; Ilkayeva, O.; Marcheva, B.; Kobayashi, Y.; Omura, C.; et al. Circadian clock NAD+ cycle drives mitochondrial oxidative metabolism in mice. *Science* **2013**, doi:10.1126/science.1243417.
122. Ying, W. NAD + /NADH and NADP + /NADPH in Cellular Functions and Cell Death: Regulation and Biological Consequences. *Antioxid. Redox Signal.* **2008**, doi:10.1089/ars.2007.1672.
123. Davila, A.; Liu, L.; Chellappa, K.; Redpath, P.; Nakamaru-Ogiso, E.; Paolella, L.M.; Zhang, Z.; Migaud, M.E.; Rabinowitz, J.D.; Baur, J.A. Nicotinamide adenine dinucleotide is transported into mammalian mitochondria. *Elife* **2018**, doi:10.7554/eLife.33246.
124. Roberts, J.H.; Stark, P.; Giri, C.P.; Smulson, M. Cytoplasmic poly(ADP-ribose) polymerase during the HeLa cell cycle. *Arch. Biochem. Biophys.* **1975**, doi:10.1016/0003-9861(75)90037-5.
125. Burzio, L.O.; Sáez, L.; Cornejo, R. Poly (ADP-ribose) synthetase activity in rat testis mitochondria. *Biochem. Biophys. Res. Commun.* **1981**, doi:10.1016/0006-291X(81)91702-2.
126. Williams, E.G.; Wu, Y.; Wolski, W.; Kim, J.Y.; Lan, J.; Hasan, M.; Halter, C.; Jha, P.; Ryu, D.; Auwerx, J.; et al. Quantifying and Localizing the Mitochondrial Proteome Across Five Tissues in A Mouse Population. *Mol. Cell. Proteomics* **2018**, doi:10.1074/mcp.RA118.000554.
127. Ahuja, N.; Schwer, B.; Carobbio, S.; Waltregny, D.; North, B.J.; Castronovo, V.; Maechler, P.; Verdin, E. Regulation of insulin secretion by SIRT4, a mitochondrial ADP-ribosyltransferase. *J. Biol. Chem.* **2007**, doi:10.1074/jbc.M705488200.
128. Niere, M.; Mashimo, M.; Agledal, L.; Dölle, C.; Kasamatsu, A.; Kato, J.; Moss, J.; Ziegler, M. ADP-ribosylhydrolase 3 (ARH3), not poly(ADP-ribose) glycohydrolase (PARG) isoforms, is responsible for degradation of mitochondrial matrix-associated poly(ADP-ribose). *J. Biol. Chem.* **2012**, doi:10.1074/jbc.M112.349183.
129. Neuvonen, M.; Ahola, T. Differential Activities of Cellular and Viral Macro Domain Proteins in Binding of ADP-Ribose Metabolites. *J. Mol. Biol.* **2009**, doi:10.1016/j.jmb.2008.10.045.
130. Richter, C.; Winterhalter, K.H.; Baumhuter, S.; Lotscher, H.R.; Moser, B. ADP-ribosylation in inner membrane of rat liver mitochondria. *Proc. Natl. Acad. Sci.* **2006**, doi:10.1073/pnas.80.11.3188.
131. Schwer, B.; North, B.J.; Frye, R.A.; Ott, M.; Verdin, E. The human silent information regulator (Sir)2 homologue hSIRT3 is a mitochondrial nicotinamide adenine dinucleotide-dependent deacetylase. *J. Cell Biol.* **2002**, doi:10.1083/jcb.200205057.
132. Choudhary, C.; Weinert, B.T.; Nishida, Y.; Verdin, E.; Mann, M. The growing landscape of lysine acetylation links metabolism and cell signalling. *Nat. Rev. Mol. Cell Biol.* **2014**, doi:10.1038/nrm3841.
133. Onyango, P.; Celic, I.; McCaffery, J.M.; Boeke, J.D.; Feinberg, A.P. SIRT3, a human SIR2 homologue, is an NAD- dependent deacetylase localized to mitochondria. *Proc. Natl. Acad. Sci.* **2002**, doi:10.1073/pnas.222538099.
134. Martello, R.; Leutert, M.; Jungmichel, S.; Bilan, V.; Larsen, S.C.; Young, C.; Hottiger, M.O.; Nielsen, M.L. Proteome-wide identification of the endogenous ADP-ribosylome of mammalian cells and tissue. *Nat. Commun.* **2016**, doi:10.1038/ncomms12917.
135. Rossi, M.N.; Carbone, M.; Mostocotto, C.; Mancone, C.; Tripodi, M.; Malone, R.; Amati, P. Mitochondrial localization of PARP-1 requires interaction with mitofilin and is involved in the maintenance of mitochondrial DNA integrity. *J. Biol. Chem.* **2009**, doi:10.1074/jbc.M109.025882.
136. Lai, Y.; Chen, Y.; Watkins, S.C.; Nathaniel, P.D.; Guo, F.; Kochanek, P.M.; Jenkins, L.W.; Szabó, C.; Clark, R.S.B. Identification of poly-ADP-ribosylated mitochondrial proteins after traumatic brain injury. *J. Neurochem.* **2008**, doi:10.1111/j.1471-4159.2007.05114.x.
137. Du, L.; Zhang, X.; Han, Y.Y.; Burke, N.A.; Kochanek, P.M.; Watkins, S.C.; Graham, S.H.; Carcillo, J.A.; Szabó, C.; Clark, R.S.B. Intra-mitochondrial poly(ADP-ribosylation) contributes to NAD+ depletion and cell death induced by oxidative stress. *J. Biol. Chem.* **2003**, doi:10.1074/jbc.M301295200.
138. Pankotai, E.; Lacza, Z.; Murányi, M.; Szabó, C. Intra-mitochondrial poly(ADP-ribosyl)ation: Potential role for alpha-ketoglutarate dehydrogenase. *Mitochondrion* **2009**, doi:10.1016/j.mito.2009.01.013.
139. Módis, K.; Gerö, D.; Erdélyi, K.; Szoleczky, P.; Dewitt, D.; Szabo, C. Cellular bioenergetics is regulated by PARP1 under resting conditions and during oxidative stress. *Biochem. Pharmacol.* **2012**, doi:10.1016/j.bcp.2011.12.014.
140. Brunyanszki, A.; Olah, G.; Coletta, C.; Szczesny, B.; Szabo, C. Regulation of Mitochondrial Poly(ADP-Ribose) Polymerase Activation by the -Adrenoceptor/cAMP/Protein Kinase A Axis during Oxidative Stress. *Mol. Pharmacol.* **2014**, doi:10.1124/mol.114.094318.
141. Yu, S.W.; Wang, H.; Poitras, M.F.; Coombs, C.; Bowers, W.J.; Federoff, H.J.; Poirier, G.G.; Dawson, T.M.; Dawson, V.L. Mediation of poty(ADP-ribose) polymerase-1 - Dependent cell death by apoptosis-inducing factor. *Science* **2002**, doi:10.1126/science.1072221.
142. Cipriani, G.; Rapizzi, E.; Vannacci, A.; Rizzuto, R.; Moroni, F.; Chiarugi, A. Nuclear poly(ADP-ribose) polymerase-1 rapidly triggers mitochondrial dysfunction. *J. Biol. Chem.* **2005**, doi:10.1074/jbc.M414526200.
143. Poitras, M.F.; Koh, D.W.; Yu, S.W.; Andrabi, S.A.; Mandir, A.S.; Poirier, G.G.; Dawson, V.L.; Dawson, T.M. Spatial and functional relationship between poly(ADP-ribose) polymerase-1 and poly(ADP-ribose) glycohydrolase in the brain. *Neuroscience* **2007**, doi:10.1016/j.neuroscience.2007.04.062.
144. Lapucci, A.; Pittelli, M.; Rapizzi, E.; Felici, R.; Moroni, F.; Chiarugi, A. Poly(ADP-ribose) Polymerase-1 Is a Nuclear Epigenetic Regulator of Mitochondrial DNA Repair and Transcription. *Mol. Pharmacol.* **2011**, doi:10.1124/mol.110.070110.
145. Druzhyna, N.; Smulson, M.E.; LeDoux, S.P.; Wilson, G.L. Poly(ADP-ribose) polymerase facilitates the repair of N-methylpurines in mitochondrial DNA. *Diabetes* **2000**, doi:10.2337/diabetes.49.11.1849.
146. Ueda, K.; Oka, J.; Narumiya, S.; Miyakawa, N.; Hayaishi, O. Poly ADP-ribose glycohydrolase from rat liver nuclei, a novel enzyme degrading the polymer. *Biochem. Biophys. Res. Commun.* **1972**, doi:10.1016/S0006-291X(72)80169-4.
147. Mashimo, M.; Bu, X.; Aoyama, K.; Kato, J.; Ishiwata-Endo, H.; Stevens, L.A.; Kasamatsu, A.; Wolfe, L.A.; Toro, C.; Adams, D.; et al. PARP1 inhibition alleviates injury in ARH3-deficient mice and human cells. *JCI Insight* **2019**, doi:10.1172/jci.insight.124519.
148. Danhauser, K.; Alhaddad, B.; Makowski, C.; Piekutowska-Abramczuk, D.; Syrbe, S.; Gomez-Ospina, N.; Manning, M.A.; Kostera-Pruszczyk, A.; Krahn-Peper, C.; Berutti, R.; et al. Bi-allelic ADPRHL2 Mutations Cause Neurodegeneration with Developmental Delay, Ataxia, and Axonal Neuropathy. *Am. J. Hum. Genet.* **2018**, doi:10.1016/j.ajhg.2018.10.005.
149. Lattin, J.E.; Schroder, K.; Su, A.I.; Walker, J.R.; Zhang, J.; Wiltshire, T.; Saijo, K.; Glass, C.K.; Hume, D.A.; Kellie, S.; et al. Expression analysis of G Protein-Coupled Receptors in mouse macrophages. *Immunome Res.* **2008**, doi:10.1186/1745-7580-4-5.
150. Zang, L.; Xue, B.; Lu, Z.; Li, X.; Yang, G.; Guo, Q.; Ba, J.; Zou, X.; Dou, J.; Lu, J.; et al. Identification of LRP16 as a negative regulator of insulin action and adipogenesis in 3T3-L1 adipocytes. *Horm. Metab. Res.* **2013**, doi:10.1055/s-0032-1331215.
151. Li, X.; Xue, B.; Wang, X.; Sun, L.; Zhang, T.; Qu, L.; Zou, X.; Mu, Y. Reduced expression of the LRP16 gene in mouse insulinoma (MIN6) cells exerts multiple effects on insulin content, proliferation and apoptosis. *J. Huazhong Univ. Sci. Technol. - Med. Sci.* **2012**, doi:10.1007/s11596-012-0034-6.
152. Li, X.J.; Guo, Q.H.; Wang, X.; Xue, B.; Sun, L.Q.; Meng, Q.T.; Lu, J.M.; Mu, Y.M. LRP16 gene protects mouse insulinoma MIN6 cells against fatty acid-induced apoptosis through Akt/FoxO1 signaling. *Chin. Med. J. (Engl).* **2012**, doi:10.3760/cma.j.issn.0366-6999.2012.10.004.
153. Li, Y.Z.; Zhao, P.; Han, W.D. Clinicopathological significance of LRP16 protein in 336 gastric carcinoma patients. *World J. Gastroenterol.* **2009**, doi:10.3748/wjg.15.4833.
154. Brunyanszki, A.; Szczesny, B.; Virág, L.; Szabo, C. Mitochondrial poly(ADP-ribose) polymerase: The Wizard of Oz at work. *Free Radic. Biol. Med.* 2016, doi:10.1016/j.freeradbiomed.2016.02.024.
155. Zhou, H.-Z.; Swanson, R.A.; Simonis, U.; Ma, X.; Cecchini, G.; Gray, M.O. Poly(ADP-ribose) polymerase-1 hyperactivation and impairment of mitochondrial respiratory chain complex I function in reperfused mouse hearts. *Am. J. Physiol. Heart Circ. Physiol.* **2006**, doi:10.1152/ajpheart.00823.2005.
156. Mayer, P.R.; Huang, N.; Dewey, C.M.; Dries, D.R.; Zhang, H.; Yu, G. Expression, localization, and biochemical characterization of nicotinamide mononucleotide adenylyltransferase 2. *J. Biol. Chem.* **2010**, doi:10.1074/jbc.M110.178913.
157. Lau, C. The NMN/NaMN adenylyltransferase (NMNAT) protein family. *Front. Biosci.* **2009**, *14*, 410–431.
158. Zhang, T.; Berrocal, J.G.; Yao, J.; DuMond, M.E.; Krishnakumar, R.; Ruhl, D.D.; Ryu, K.W.; Gamble, M.J.; Kraus, W.L. Regulation of poly(ADP-ribose) polymerase-1-dependent gene expression through promoter-directed recruitment of a nuclear NAD + synthase. *J. Biol. Chem.* **2012**, doi:10.1074/jbc.M111.304469.
159. Hottiger, M.O. SnapShot: ADP-Ribosylation Signaling. *Mol. Cell* **2015**, doi:10.1016/j.molcel.2015.06.001.
160. Fouquerel, E.; Sobol, R.W. ARTD1 (PARP1) activation and NAD+ in DNA repair and cell death. *DNA Repair (Amst).* **2014**, doi:10.1016/j.dnarep.2014.09.004.
161. Kraus, W.L.; Hottiger, M.O. PARP-1 and gene regulation: Progress and puzzles. *Mol. Aspects Med.* **2013**, doi:10.1016/j.mam.2013.01.005.
162. Posavec Marjanović, M.; Crawford, K.; Ahel, I. PARP, transcription and chromatin modeling. *Semin. Cell Dev. Biol.* **2017**, doi:10.1016/j.semcdb.2016.09.014.
163. Jubin, T.; Kadam, A.; Gani, A.R.; Singh, M.; Dwivedi, M.; Begum, R. Poly ADP-ribose polymerase-1: Beyond transcription and towards differentiation. *Semin. Cell Dev. Biol.* **2017**, doi:10.1016/j.semcdb.2016.07.027.
164. Schiewer, M.J.; Knudsen, K.E. Transcriptional Roles of PARP1 in Cancer. *Mol. Cancer Res.* **2014**, doi:1158/1541-7786.
165. Abplanalp, J.; Hottiger, M.O. Cell fate regulation by chromatin ADP-ribosylation. *Semin. Cell Dev. Biol.* **2017**, doi:10.1016/j.semcdb.2016.09.010.
166. Chalkiadaki, A.; Guarente, L. The multifaceted functions of sirtuins in cancer. *Nat. Rev. Cancer* **2015**, doi:10.1038/nrc3985.
167. Mao, Z.; Hine, C.; Tian, X.; Van Meter, M.; Au, M.; Vaidya, A.; Seluanov, A.; Gorbunova, V. SIRT6 promotes DNA repair under stress by activating PARP1. *Science* **2011**, doi:10.1126/science.1202723.
168. Rezazadeh, S.; Yang, D.; Tombline, G.; Simon, M.; Regan, S.P.; Seluanov, A.; Gorbunova, V. SIRT6 promotes transcription of a subset of NRF2 targets by mono-ADP-ribosylating BAF170. *Nucleic Acids Res.* **2019**, doi:10.1093/nar/gkz528.
169. Darlington, G.J.; Ross, S.E.; MacDougald, O.A. The role of C/EBP genes in adipocyte differentiation. *J. Biol. Chem.* **1998**, doi:10.1074/jbc.273.46.30057.
170. Poli, V. The role of C/EBP isoforms in the control of inflammatory and native immunity functions. *J. Biol. Chem.* **1998**, doi:10.1074/jbc.273.45.29279.
171. Westmacott, A.; Burke, Z.D.; Oliver, G.; Slack, J.M.W.; Tosh, D. C/EBPα and C/EBPβ are markers of early liver development. *Int. J. Dev. Biol.* **2006**, doi:10.1387/ijdb.062146aw.
172. Tiranti, V.; Rossi, E.; Rocchi, M.; DiDonato, S.; Zuffardi, O.; Zeviani, M. The gene (nfe2l1) for human nrf-1, an activator involved in nuclear- mitochondrial interactions, maps to 7q32. *Genomics* **1995**, doi:10.1006/geno.1995.1094.
173. Hossain, M.B.; Ji, P.; Anish, R.; Jacobson, R.H.; Takada, S. Poly(ADP-ribose) polymerase 1 interacts with nuclear respiratory factor 1 (NRF-1) and plays a role in NRF-1 transcriptional regulation. *J. Biol. Chem.* **2009**, doi:10.1074/jbc.M807198200.
174. Smith, T.G.; Robbins, P.A.; Ratcliffe, P.J. The human side of hypoxia-inducible factor *Br. J. Haematol.* **2008**, doi:10.1111/j.1365-2141.2008.07029.x.
175. Hulse, M.; Caruso, L.B.; Madzo, J.; Tan, Y.; Johnson, S.; Tempera, I. Poly(ADP-ribose) polymerase 1 is necessary for coactivating hypoxia-inducible factor-1-dependent gene expression by Epstein-Barr virus latent membrane protein 1. *PLoS Pathog.* **2018**, doi:10.1371/journal.ppat.1007394.
176. Martínez-Romero, R.; Martínez-Lara, E.; Aguilar-Quesada, R.; Peralta, A.; Oliver, F.J.; Siles, E. PARP-1 modulates deferoxamine-induced HIF-1α accumulation through the regulation of nitric oxide and oxidative stress. *J. Cell. Biochem.* **2008**, doi:10.1002/jcb.21781.
177. Rahman, S.; Islam, R. Mammalian Sirt1: Insights on its biological functions. *Cell Commun. Signal.* **2011**, doi:10.1186/1478-811X-9-11.
178. Satoh, A.; Brace, C.S.; Rensing, N.; Cliften, P.; Wozniak, D.F.; Herzog, E.D.; Yamada, K.A.; Imai, S.I. Sirt1 extends life span and delays aging in mice through the regulation of Nk2 Homeobox 1 in the DMH and LH. *Cell Metab.* **2013**, doi:10.1016/j.cmet.2013.07.013.
179. Bai, P.; Canto, C.; Brunyánszki, A.; Huber, A.; Szántó, M.; Cen, Y.; Yamamoto, H.; Houten, S.M.; Kiss, B.; Oudart, H.; et al. PARP-2 regulates SIRT1 expression and whole-body energy expenditure. *Cell Metab.* **2011**, doi:10.1016/j.cmet.2011.03.013.
180. Mohamed, J.S.; Hajira, A.; Pardo, P.S.; Boriek, A.M. MicroRNA-149 inhibits PARP-2 and promotes mitochondrial biogenesis via SIRT-1/PGC-1α network in skeletal muscle. *Diabetes* **2014**, doi:10.2337/db13-1364.
181. Szántó, M.; Rutkai, I.; Hegedus, C.; Czikora, Á.; Rózsahegyi, M.; Kiss, B.; Virág, L.; Gergely, P.; Tóth, A.; Bai, P. Poly(ADP-ribose) polymerase-2 depletion reduces doxorubicin-induced damage through SIRT1 induction. *Cardiovasc. Res.* **2011**, doi:10.1093/cvr/cvr246.
182. Geng, B.; Cai, Y.; Gao, S.; Lu, J.; Zhang, L.; Zou, J.; Liu, M.; Yu, S.; Ye, J.; Liu, P. PARP-2 knockdown protects cardiomyocytes from hypertrophy via activation of SIRT1. *Biochem. Biophys. Res. Commun.* **2013**, doi:10.1016/j.bbrc.2012.11.132.
183. Iyengar, S.; Farnham, P.J. KAP1 protein: An enigmatic master regulator of the genome. *J. Biol. Chem.* **2011**, doi:10.1074/jbc.R111.252569.
184. Van Meter, M.; Kashyap, M.; Rezazadeh, S.; Geneva, A.J.; Morello, T.D.; Seluanov, A.; Gorbunova, V. SIRT6 represses LINE1 retrotransposons by ribosylating KAP1 but this repression fails with stress and age. *Nat. Commun.* **2014**, doi:10.1038/ncomms6011.
185. Cantó, C.; Sauve, A.A.; Bai, P. Crosstalk between poly(ADP-ribose) polymerase and sirtuin enzymes. *Mol. Aspects Med.* **2013**, doi:10.1016/j.mam.2013.01.004.
186. Zong, W.X.; Ditsworth, D.; Bauer, D.E.; Wang, Z.Q.; Thompson, C.B. Alkylating DNA damage stimulates a regulated form of necrotic cell death. *Genes Dev.* **2004**, doi:10.1101/gad.1199904.
187. Bai, P.; Cantó, C.; Oudart, H.; Brunyánszki, A.; Cen, Y.; Thomas, C.; Yamamoto, H.; Huber, A.; Kiss, B.; Houtkooper, R.H.; et al. PARP-1 inhibition increases mitochondrial metabolism through SIRT1 activation. *Cell Metab.* **2011**, doi:10.1016/j.cmet.2011.03.004.
188. Pirinen, E.; Cantó, C.; Jo, Y.S.; Morato, L.; Zhang, H.; Menzies, K.J.; Williams, E.G.; Mouchiroud, L.; Moullan, N.; Hagberg, C.; et al. Pharmacological inhibition of poly(ADP-ribose) polymerases improves fitness and mitochondrial function in skeletal muscle. *Cell Metab.* **2014**, doi:10.1016/j.cmet.2014.04.002.
